# Remote Assessment of Physical Function and Physical Activity in Adults With Autism Spectrum Disorder: Protocol for Feasibility, Validity, and Reliability Study

**DOI:** 10.2196/86020

**Published:** 2026-08-31

**Authors:** Annabel Nunez-Gaunaurd, Melissa M Tovin, Melissa Hale, Tan Li, Anjana Bhat

**Affiliations:** 1 Department of Physical Therapy Nicole Wertheim College of Nursing & Health Sciences Florida International University Miami, FL United States; 2 Department of Physical Therapy Miller School of Medicine University of Miami Miami, FL United States; 3 Division Director, Child and Family Services College of Arts and Sciences University of Miami Miami, FL United States; 4 Department of Biostatistics Stempel College of Public Health Florida International University Miami, FL United States; 5 Department of Physical Therapy College of Health Sciences University of Delaware Newark, DE United States

**Keywords:** autism spectrum disorder, physical activity, co-participation, family support, autism in adulthood, telehealth, wearable devices, physical function, remote assessment, health disparities, minority health, adult autism, protocol study, cross-sectional design, patient-reported outcomes, motor impairments, rehabilitation, digital health

## Abstract

**Background:**

Adults with autism spectrum disorder (ASD) are at increased risk for chronic health conditions and reduced quality of life (QoL), often linked to lower physical activity (PA), motor impairments, and barriers to health care access. Although communication and social differences in ASD are well documented, motor impairments such as deficits in coordination, balance, and gait remain understudied in adulthood despite their relevance to fall risk and PA participation. Telehealth and wearable technologies may improve access to physical therapy assessment and remote monitoring of physical function in this underserved population.

**Objective:**

This pilot study aims to evaluate the feasibility, construct validity, and rating reliability of remotely administered physical function and balance assessments in adults with ASD and to examine racial and ethnic disparities and associations among physical function, PA, fall risk, and QoL.

**Methods:**

This pilot study uses a 2-phase, cross-sectional, noninterventional design with remote data collection in legally independent adults aged 18-59 years with ASD who were recruited through the Simons Powering Autism Research and Knowledge (SPARK) registry. Participants complete self-report questionnaires; 2 weeks of wearable step monitoring using a Fitbit Inspire 3 (Google LLC) device; and, for a randomized subset of 25 participants, 1 remote, telehealth-based physical function assessment session. Remote performance-based measures include the Five Times Sit-to-Stand, 30-second Sit-to-Stand, standing balance under eyes open and eyes closed conditions, and single-leg stance under eyes open and eyes closed conditions. Construct validity of the Patient-Reported Outcomes Measurement Information System (PROMIS) physical function measures will be examined using hypothesis testing with related indicators of health and functioning, including steps per day, lower-extremity functional outcome surveys, fall risk screening surveys, and comorbidity burden. Interrater and intrarater reliability of remote performance scoring will be evaluated from video-recorded sessions scored by 6 raters using intraclass correlation coefficients. Secondary analyses will examine racial and ethnic disparities in physical function, PA, and QoL, as well as associations among these variables and moderation by race and ethnicity.

**Results:**

Study funding was available from August 2024 to May 2025. Recruitment occurred from December 2024 to March 2025, and final data collection was completed in May 2025. By May 2025, 155 adults with ASD had been recruited, of whom 65.2% were female and 77% identified as Black or Hispanic. Data analysis is projected to be completed in July 2026, and submission of the primary results manuscript is anticipated in August 2026.

**Conclusions:**

This protocol describes a pilot study designed to evaluate the feasibility, construct validity, and equity relevance of remotely administered physical function assessments in adults with ASD. If supported, the findings may provide preliminary evidence for more accessible telehealth-based assessment approaches and inform larger studies on physical functioning, PA, and QoL in this population.

**International Registered Report Identifier (IRRID):**

DERR1-10.2196/86020

## Introduction

### Background

Autism spectrum disorder (ASD) is a lifelong neurodevelopmental condition characterized by persistent differences in social communication and interaction, alongside restricted and repetitive patterns of behavior and interests [1]. Beyond these core diagnostic features, many autistic individuals experience clinically meaningful motor impairments, including reduced coordination, poor balance, atypical gait, and diminished muscle strength [2-5]. These impairments often persist into adulthood and across the life course [6-9], yet they remain relatively underrecognized in adult ASD research and clinical care. Motor limitations may reduce participation in physical activity (PA), increase sedentary behavior, and contribute to an elevated risk of falls, obesity, and chronic health conditions, ultimately affecting overall functioning and quality of life (QoL).

Despite their relevance, motor impairments and physical function remain underresearched in adults with ASD. These domains are not included in diagnostic criteria, are inconsistently assessed in clinical practice, and are infrequently addressed through movement-based rehabilitation strategies [2,10,11]. The limited use of standardized, objective measures of physical function in autistic adults also restricts the ability to evaluate intervention effects, monitor change over time, and tailor supports to individual needs [10]. This gap underscores a pressing need for accessible, valid, and reliable methods to assess physical function and motor performance in adults with ASD.

Barriers to health care access further complicate the evaluation and management of physical function in this population. Adults with ASD often encounter communication challenges, sensory sensitivities, anxiety, and limited provider familiarity with adult ASD, all of which may interfere with effective assessment and treatment [12-14]. These barriers are often compounded for racial and ethnic minority populations, who may also experience delayed diagnosis, underrecognition of co-occurring conditions, and structural inequities in care [15-17]. Additional barriers related to transportation, geography, financial constraints, and health system complexity may further reduce participation in clinic-based care [18-20]. Telehealth-based approaches may help address these barriers by reducing logistical burdens, minimizing sensory and social stressors, and expanding participation in both research and clinical services.

Telehealth has emerged as a practical modality for functional assessment in both clinical and research settings. Synchronous telehealth allows for real-time administration and observation of performance-based measures, whereas asynchronous video review allows repeated observation, more detailed scoring, and evaluation of interrater and intrarater reliability [21-24].

Together, these approaches may improve accessibility while preserving observational rigor. However, although telehealth-based rehabilitation and remote monitoring are increasingly used, standardized evaluations of physical function in adults with ASD remain limited, and psychometrically supported remote assessment methods for this population are largely absent from the literature [2,10,11].

This protocol addresses this gap by examining the feasibility, construct validity, and rating reliability of remotely administered physical function assessments in adults with ASD. Consistent with the analytic plan, the study evaluates the construct validity of patient-reported physical function using related indicators of health and functioning, including PA, lower-extremity function, fall risk, and comorbidity burden, while also examining remote performance-based measures as exploratory complementary indicators of physical function. The protocol additionally investigates racial and ethnic disparities in physical function, PA, fall risk, and QoL and evaluates whether the relationships among physical function, PA, and QoL differ by race and ethnicity. By focusing on telehealth-delivered assessments and equitable inclusion of racial and ethnic minority participants, this study seeks to inform more accessible and inclusive approaches to physical function assessment in adults with ASD.

### Guiding Theoretical Model

This study is guided by the Model of Healthcare Disparities and Disability (MHDD**)** [25], which extends disability-focused frameworks by explicitly incorporating health care access, system-level barriers, and contextual influences on functioning and health outcomes.

**Figure 1:**
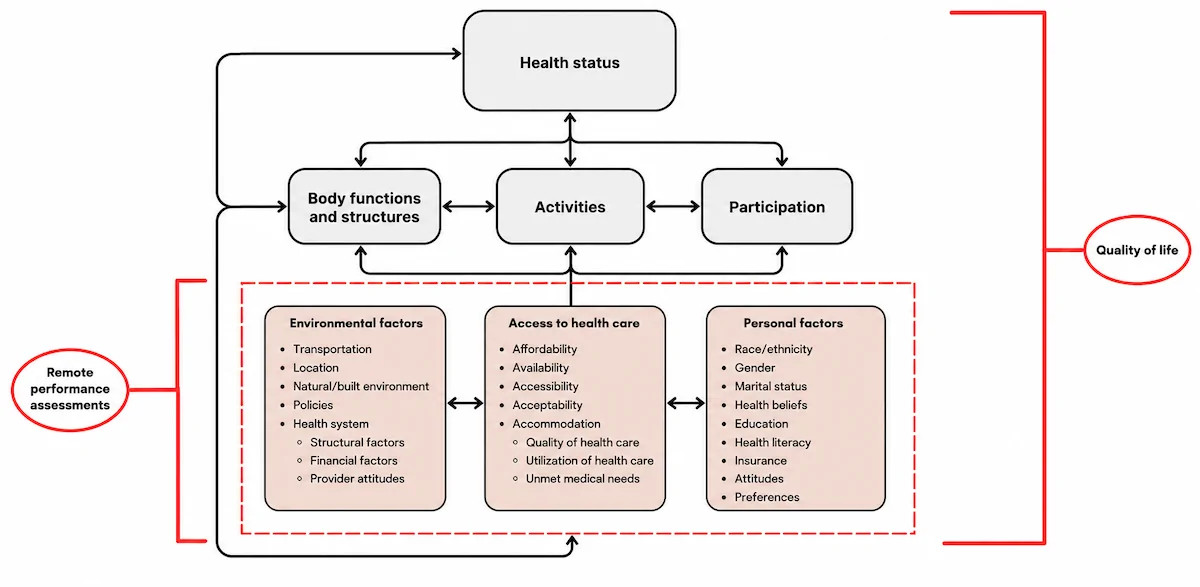
This figure illustrates the theoretical framework informing the study, adapted from the original Model of Healthcare Disparities and Disability (MHDD). The red rectangle highlights the specific focus of this research: contextual barriers to health care access for individuals with disabilities. Remotely administered performance-based measures are designed to overcome these barriers to improve access and quality of health care, support optimal functioning, and enhance quality of life.

Whereas the International Classification of Functioning, Disability, and Health (ICF) [26] recognizes the interaction among body functions, activity, participation, and contextual factors, the MHDD more directly emphasizes how mismatches between individual characteristics and environmental or health system conditions can contribute to reduced access to care, diminished functioning, and poorer health outcomes among people with disabilities (see Figure 1**)** [25]. Within this framework, remotely administered physical function assessment is conceptualized as a strategy to reduce environmental and health care access barriers that may disproportionately affect adults with ASD, particularly those from underserved or racial and ethnic minoritized groups. Telehealth may support participation by allowing assessment to occur in a familiar environment and by reducing transportation-related burden, clinic-related stress, and sensory overload. In this way, remote physical function assessment is positioned not only as a methodological innovation but also as a potential mechanism for improving access to health services relevant to physical functioning.

Although QoL is not represented as a discrete construct within the MHDD, it is closely related to the model’s emphasis on functioning, participation, and contextual fit of the individual. Inadequate access to appropriate health services, environmental barriers, and poor alignment between individual needs and care delivery may negatively influence functioning and participation, thereby affecting QoL. In this protocol, QoL is conceptualized as an important downstream outcome linked to physical function, PA, health burden, and access-related factors. The MHDD provides a useful framework for understanding how remotely delivered physical function assessments may support more equitable health evaluations and, ultimately, more responsive care for adults with ASD.

### Objectives

This paper presents the protocol for a pilot study designed to evaluate the feasibility, construct validity, and rating reliability of remotely administered physical function assessments in adults with ASD, with attention to racial and ethnic equity in participation and outcomes.

This study has three primary aims:

1. To evaluate the feasibility, construct validity, and rating reliability of remote physical function assessment in adults with ASD. Construct validity will be examined primarily through hypothesis testing of the Patient-Reported Outcomes Measurement Information System–Physical Function (PROMIS-PF) measure against related indicators of health and functioning, including habitual PA, lower-extremity functional outcome surveys, fall risk screening surveys, and comorbidity burden. Remote performance-based measures will also be examined as exploratory complementary indicators of physical function. Interrater and intrarater reliability of remote performance scoring will be evaluated using video-recorded assessment sessions scored by multiple raters.
2. To examine racial and ethnic disparities in physical function, PA, fall risk, and QoL among adults with ASD. This objective is intended to generate preliminary evidence regarding differences in health and functioning across racial and ethnic groups within a telehealth-based study sample.
3. To examine associations among physical function, PA, personal and environmental factors, and QoL, and to evaluate whether these relationships vary by race and ethnicity. This objective will assess whether physical function and PA are associated with QoL and whether race/ethnicity moderates these relationships.

This protocol is intended to provide foundational evidence for more accessible, equitable, and scalable approaches to physical function assessment in adults with ASD. If the planned hypotheses are supported, the findings may inform future larger-scale studies and telehealth-based rehabilitation strategies aimed at improving physical function, PA, and QoL in this population.

## Methods

### Study Design and Procedures

This pilot study will use a 2-phase, cross-sectional, noninterventional design with remote data collection in adults with ASD, with targeted recruitment of individuals from underrepresented racial and ethnic groups. This study uses a nested design. The full sample of participants will complete the self-report and wearable activity monitoring components of the study. Within this overall sample, a randomized subset of 25 participants will also complete Phase 1, which consists of a remote telehealth-based performance assessment session. Accordingly, Phase 1 participants are included within the overall study cohort and are not treated as a separate sample.

### Ethical Considerations

This study was approved by the Institutional Review Board of Florida International University (Protocol #IRB-24-0230-AM5; TOPAZ Reference #113500). Electronic informed consent will be obtained from all participants prior to study participation. Participant data will be deidentified and handled in accordance with institutional and applicable regulatory requirements for privacy and confidentiality. Data were securely stored and managed using REDCap (Vanderbilt University).

### Study Population

Eligible participants will be adults with a self-reported professional diagnosis of ASD registered in the Simons Powering Autism Research and Knowledge (SPARK) registry. SPARK is a nationwide database of individuals residing in the United States and will serve as the recruitment source for this study [27]. The registry has demonstrated high diagnostic validity (98.8% agreement rate) [28] and includes over 13,000 adults, approximately 55% of whom are legally independent. Participants will be required to have access to a personal computing device with internet connectivity and the ability to complete remote assessments.

### Inclusion/Exclusion Criteria

The inclusion criteria are legally independent adults with ASD aged 18-59 years, with a previous professional diagnosis or educational classification of ASD, autism/autistic disorder, Asperger syndrome, or pervasive developmental disorder not otherwise specified. Participants will be registered with the SPARK network, and their available registry data must include age at registration, sex, race, ethnicity, professional or professionals who provided the ASD diagnosis, age at initial diagnosis, lifetime receipt of services, participation in an individualized education program or other ASD-specific programs, and autism severity as measured by the Social Responsiveness Scale (SRS). Persons will be excluded from the study if they have any open wounds, fractures, severe visual or hearing impairments, or health conditions that prevent them from standing up from a chair or standing on either leg or that preclude safe participation in remote assessment.

### Recruitment

To target the recruitment of adults with ASD from underrepresented racial and ethnic groups, we will implement a multipronged strategy in collaboration with the SPARK Research Match service. Research Match, an integrated service of the Simons Foundation Autism Research Initiative (SFARI), enables approved investigators to recontact SPARK participants for new studies. The platform supports both remote and in-person research and streamlines enrollment by integrating invitation, eligibility screening, and survey access into a single system. Recruitment will be conducted via email outreach through SPARK’s “batched recruitment” infrastructure, which allows invitations to be distributed in waves to selected subsets of eligible participants until the enrollment targets are met. Stratified sampling will be used to ensure the representation of Black/African American and Hispanic/Latino participants, with random selection continuing until quotas of 50 participants per group are achieved.

Eligible individuals will be invited to review and electronically sign informed consent forms. We plan to enroll a total of 155 participants. All enrolled participants will complete the self-report and wearable activity monitoring components of the study. Within this overall sample, a randomized subset of 25 participants will be invited to complete the Phase 1 remote telehealth-based performance assessment session. Thus, the Phase 1 subset is nested within the full study sample.

### Design of Functional Assessment and Self-Report Surveys Testing

**Figure 2:**
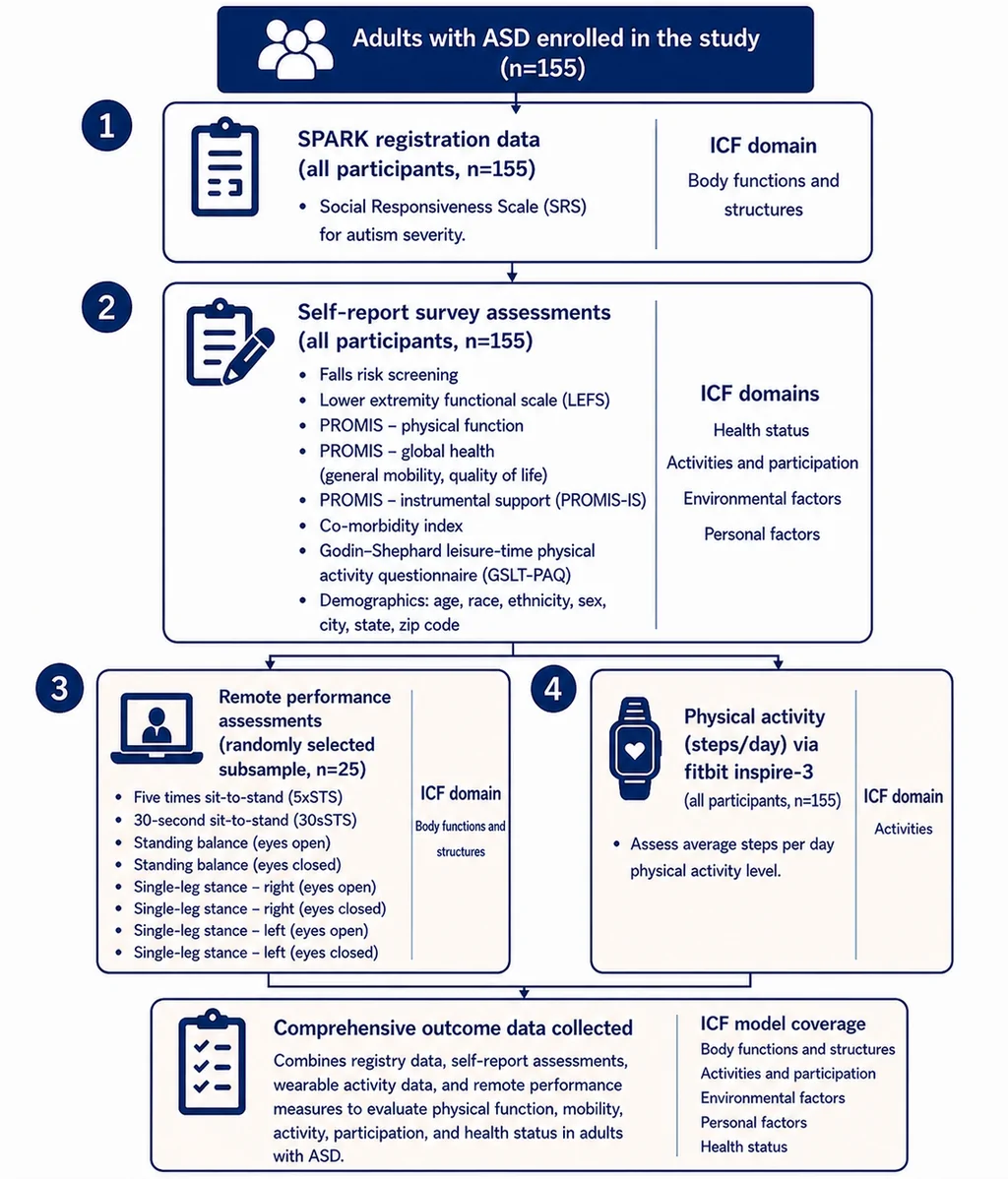
Sequence of outcome measurement. ASD: autism spectrum disorder; ICF: International Classification of Functioning, Disability, and Health; PROMIS: Patient-Reported Outcomes Measurement Information System; SPARK: Simons Powering Autism Research and Knowledge.

The study includes three main forms of data collection: (1) telehealth-delivered performance-based physical function assessments, (2) self-report survey measures, and (3) wearable activity monitoring of daily step counts. Measures were selected following a review of available assessments with an emphasis on usability, accessibility, and relevance for adults with ASD, including consideration of the sensory, cognitive, and motor features that may influence remote assessment participation and performance. A summary of health outcome measures and related International Classification of Functioning, Disability and Health domains is provided in Table 1, and the sequence of outcome measure assessments is provided in Figure 2.

**Table 1:** Outcome measures.

Outcome instrument			Outcomes assessed		ICF^a^-Meade conceptual model domain
Remote performance assessments					
	5xSTS^b^ [29,30]	Lower extremity strength and functional mobility		Body functions and structures	
	30sSTS^c^ [31,32]	Lower body strength and endurance validated for use in remote assessments		Body functions and structures	
	Standing balance (eyes open/closed) [33,34]	Static balance under eyes open and closed conditions; derived from BBS^d^ items commonly used in neurological populations		Body functions and structures	
	SLS-R/L^e^ eyes open, and eyes closed [35]	Balance and vestibular function assessed separately for right and left limbs		Body functions and structures	
	Physical activity (steps/day) Fitbit Inspire 3 (Google LLC) [36-38]	Objective measurement of daily physical activity via step count		Activities	
Self-report survey assessments					
	STEADI^f^-Falls Risk Screening [39]	Multidimensional fall risk screening (mobility, physical function, medical history, environmental hazards, and cognition/mental health)		Health status	
	LEFS^g^ [40,41]	20-item self-report measure of functional status in individuals with lower limb musculoskeletal conditions		Activities/participation	
	PROMIS^h^-PF^i^ [42,43]	Self-reported ability to perform physical activities (short form)		Activities/participation	
	PROMIS-Global Health (general mobility and QoL^j^) [44]	General health status, including physical function, pain, fatigue, emotional distress, and social well-being		Activities/participation; personal factors; QoL	
	PROMIS-IS^k^ [45]	Perceived availability of social and IS		Environmental factors	
	Co-Morbidity Index [46]	Impact of chronic health conditions on functional outcomes		Personal factors	
	GSLT-PAQ^l^ [47]	Self-reported leisure-time physical activity participation		Activities/participation	
	Demographics: age, race, ethnicity, sex, city, state, ZIP code	Age, race, ethnicity, gender, and geographic characteristics (city, state, and ZIP code)		Personal/environmental factors	
SPARK^m^ registration data					
	Social Responsiveness Scale-Autism Severity [48]	Quantitative measure of social impairment associated with ASD^n^ relatively stable trait over time		Body functions activities and participation	

^a^ICF: International Classification of Functioning, Disability, and Health.

^b^5xSTS: Five Times Sit-to-Stand.

^c^30sSTS: 30-Second Sit-to-Stand.

^d^BBS: Berg Balance Scale.

^e^SLS-R/L: right/left single-leg stance.

^f^STEADI: Stopping Elderly Accidents, Deaths, and Injuries.

^g^LEFS: Lower Extremity Functional Scale

^h^PROMIS: Patient-Reported Outcomes Measurement Information System.

^i^PF: physical function.

^j^QoL: quality of life.

^k^IS: instrumental support.

^l^GSLT-PAQ: Godin-Shephard Leisure-Time Physical Activity Questionnaire.

^m^SPARK: Simons Powering Autism Research and Knowledge.

^n^ASD: autism spectrum disorder.

Study activities are organized into 2 linked phases within a nested design. Phase 1 focuses on the remote telehealth-based performance assessment component and is completed by a randomized subset of 25 participants drawn from the overall study cohort. Phase 2 includes the full study sample and comprises self-report survey measures and wearable activity monitoring. Thus, all enrolled participants complete the Phase 2 components, whereas the Phase 1 subset additionally completes the remote performance-based assessment protocol.

All participants will complete online survey measures using REDCap and will be mailed a Fitbit Inspire 3 (Google LLC) device to wear for 2 weeks. Daily step-count data will be downloaded through the Fitabase (Small Steps Labs, LLC) platform. Completion of self-report surveys and wearable-monitor compliance for 2 weeks is required for participation in the full study sample. For the nested Phase 1 subset, participation also includes one 60-minute telehealth-based physical function assessment session.

### Phase 1: Validation of Remote Functional Assessments

In Phase 1, a randomized selection of 25 participants will undergo remote telehealth-based physical function assessments via secure platforms using their personal computing devices. The telehealth assessment includes a complete battery of physical function, including the Five Times Sit-to-Stand (5xSTS), 30-second Sit-to-Stand (30sSTS), standing balance (eyes open [EO] and eyes closed [EC]), and Single-Leg Stance (SLS; EO and EC). All assessments will be recorded to support intra- and interrater reliability analyses across 6 independent raters. Feasibility will be evaluated based on completion rates, participant feedback, and technical challenges.

### Rater Training

To assess interrater reliability of video-recorded remote assessments, the primary rater, a licensed physical therapist (PT) with expertise in functional outcome measures and experience working with individuals with ASD, will train 4 second-year and 3 third-year Doctor of Physical Therapy (DPT) students from the same academic institution. Training will include structured instruction on scoring procedures, video-based calibration sessions, and a formal agreement check-off to ensure consistency prior to data collection. All raters will also receive specialized training in safety monitoring tailored to the ASD population.

Calibration will be conducted using 3 mock video-recorded assessments representing the study performance battery. To qualify for independent scoring, each student rater must achieve at least 80% agreement with the primary rater across scored items during the formal check-off. Raters who do not meet this criterion will receive additional feedback and retraining and will repeat calibration until the required agreement level is achieved before scoring study recordings independently.

Although licensed PTs typically administer remote functional assessments, supervised DPT students may contribute as part of their clinical training. Their involvement in this study highlights the potential for integrating student participation into telehealth-based evaluations and may support broader implementation in educational and clinical settings.

### Preremote Session Instructions

Prior to the scheduled session, participants will receive an email with detailed instructions to prepare their environment and equipment. Participants will also be encouraged to log into Zoom (Zoom Video Communications, Inc) a few minutes early to troubleshoot any technical issues. Additionally, given the unique sensory, cognitive, and motor coordination profiles of adults with ASD, additional safety measures will be implemented, and the email will include the following guidance and recommendations when possible:

- Chair requirements: a sturdy chair with a seat height of approximately 43-45 cm (knee height), equipped with armrests and a backrest.
- Open space: a clear, unobstructed area free of tripping hazards to safely perform the tests.
- Quiet environment: a distraction-free setting to support concentration and communication.
- Camera setup: the device camera should be positioned to capture the participants’ full body during both seated and standing movements.

### Day of Remote Session Safety Check

Given the unique sensory, cognitive, and motor coordination profiles of adults with ASD, additional safety measures will be implemented. Once the participant and rater have successfully logged into the secure Zoom video platform, the rater will greet the participant; confirm readiness; and identify any sensory sensitivities, communication preferences, or physical limitations that may affect test performance. All raters will ensure the following safety measures and instructions are provided to the study participant to optimize remote testing sessions: (1) new or recent medical conditions, injuries or changes in physical status that may affect test performance; (2) quiet environment, when possible, the study participant will be encouraged to choose a space that is quiet and free from distractions to help with focus during the evaluation; and (3) computer setup, participants will be encouraged to position a computer or device so that the rater can see their entire body during the tests. The camera should also be at a height that allows the rater to see them sitting and standing. If necessary, the rater will use their own device to demonstrate body positioning and spacing expected of the participant. Testing sessions will be paused or discontinued if participants exhibit safety concerns, including but not limited to dizziness, chest pain, shortness of breath, loss of balance, or expressed discomfort. The trained assessor conducting the session will make the determination to pause or terminate testing based on these criteria.

### Remote Video Assessment

Each participant will complete a remote telehealth-based performance assessment session, which will be video-recorded. Following the safety check, the rater may begin the remote-based performance assessment. The evaluation will include standardized performance-based functional assessments, including the 5xSTS test, 30sSTS, modified Berg Balance Scale (BBS) items (standing EC/EO), and SLS test. These assessments will be conducted in real time by a trained rater and are designed to measure lower extremity strength, balance, and functional mobility. The rater will provide the following to optimize testing procedures. First, clear instructions and demonstrations to all study participants will be given. All test procedures will be explained using simple, direct language. Visual demonstrations will be provided, and participants will be allowed to practice each movement before formal testing begins. Second, real-time monitoring will be used, ensuring that the rater continuously monitors participant posture, movement quality, and signs of distress or fatigue. Testing will be paused or discontinued if safety concerns arise. Third, rest breaks will be given. The participants will be offered rest breaks between assessments and trial sets to prevent fatigue and accommodate sensory regulation needs. Video recordings will be used exclusively for evaluation of interrater and intrarater reliability of rater scoring; no repeat participant assessments will be conducted. Interrater reliability will be evaluated by having 6 independent raters score the same recordings. Intrarater reliability will be evaluated by having each rater re-score the same recordings after a 2-5-day interval.

### Performance-Based Outcome Measures

#### 5xSTS

This test assesses lower extremity strength and functional mobility. It has demonstrated appropriate feasibility, strong reliability, and validity in both in-person and remote formats across various populations, including older adults, individuals with chronic conditions, and individuals with cancer [29,30]. Participants will be instructed to sit with their arms folded across their chest and back against the chair. They will be asked to stand up and sit down 5 times as quickly as possible. The time to complete the 5 repetitions will be recorded using a stopwatch. A shorter completion time indicates better lower limb strength [30].

#### 30sSTS

A measure of lower body strength and endurance, the 30sSTS has been validated for use in remote assessments and is sensitive to functional limitations in diverse populations [31,32]. Participants will be instructed to complete as many full stands as possible within 30 seconds. The chair will be placed against a wall to prevent movement. Participants will sit in the middle of the chair with feet shoulder-width apart and arms crossed at the chest. A demonstration will be provided, and participants will practice 1-2 repetitions before the test. If arm use is required, the score will be recorded as zero. The total number of correctly executed stands will be counted silently by the assessor. Partial or incorrect stands will not be included in the final score.

#### Standing Balance Tests (Bipedal Stance, EO/EC)

Participants will perform bipedal stance with EO and EC to assess static balance. This assessment is often associated with one of the 14 testing items from the BBS, a widely used tool to assess balance, especially in patients with neurological diseases [34]. The BBS contains and commonly estimates the risk of falls. It is important to determine the suitability of the BBS, which is frequently used in clinical practice, for telehealth assessment.

Participants will be asked to stand barefoot (if tolerated), with their feet side-by-side and arms crossed over their chest. For EO trials, participants will focus on a visual target at eye level. Timing will begin when the tester says “Go.” For EC trials, participants will close their eyes upon instruction after they are positioned. Each trial will end if the participant moves the feet, uncrosses/moves the arms, opens the eyes during EC, loses balance/uses external support, or reaches the 45-second maximum. Participants will complete 2 trials with EO and 2 trials with EC, alternating conditions to reduce fatigue and habituation. These tests are commonly used in clinical settings and have demonstrated feasibility for remote administration [33].

#### SLS-Right/Left, EO/EC

The timed unipedal stance test (EO/EC) evaluates balance and vestibular function. It has excellent interrater reliability and established normative values [35]. Unipedal balance (30-second SLS) was found to be reliable and valid for in-person functional outcomes assessment of lower extremity strength and balance and has recently been examined for remote assessments [35]. Participants will be asked to stand barefoot (if tolerated) on one limb, with the opposite foot raised near but not touching the stance limb. The arms will be crossed over the chest, and the participants will focus on a visual target at eye level. Timing will begin when the foot is lifted and will end upon any of the following: arm movement away from the chest, foot contact, stance-foot repositioning, loss of balance/use of external support, opening the eyes during EC, or reaching the 45-second maximum. SLS will be performed on both the right and left legs under both EO and EC conditions. Each participant will complete 2 trials per leg per condition (EO: 2 trials right and 2 trials left; EC: 2 trials right and 2 trials left), alternating EO and EC conditions. The order of testing (starting leg and/or condition) will be randomized, and 1-2 minutes of rest will be provided between trial sets. The best time for each leg and condition will be recorded; for analyses, the primary outcome for EO and EC will be the best (longest) time across both legs and all trials (capped at 45 seconds). Average times across trials may also be summarized descriptively.

#### Free-Living Daily PA (Steps/Day)

Steps per day will be assessed using a Fitbit Inspire 3 wearable device, a commercially available wearable activity tracker that records the total amount of steps/day [37]. PA tracking with consumer-based wearables has a relatively high correlation with research-grade accelerometers [49]. At least 7 days of walking activity data (5 weekdays and 2 weekend days) with at least 10 waking hours will be required for data analysis [36]. Additionally, the Fitbit can upload real-time step data wirelessly to a web-based database for longitudinal monitoring and data storage (Fitabase) [38]. These data together with the Godin-Shephard Leisure-Time Physical Activity Questionnaire (GSLT-PAQ; described below) will provide a more holistic assessment of PA, as the GSLT-PAQ offers a more qualitative examination, requiring an awareness and perception of one’s PA level.

### Phase 2: Self-Report Survey Assessments

Identification and examination of demographics, perceived physical function, fall risk, health-related QoL, and PA will be conducted in Phase 2 of this study. A summary of the health outcome measures and related ICF domains is presented in Table 1.

### Self-Report Surveys

#### Demographic Characteristics

Demographic variables, including race and ethnicity, state, ZIP code, and sex, will be collected via surveys to support stratified and moderation analyses.

#### SRS-Autism Severity

Autism severity will be assessed using the SRS data available from participants’ enrollment in the SPARK cohort. The SRS is a widely used and validated screening measure of autism-related social impairment [48]. Although prior work suggests some temporal variability in SRS scores [50], with adolescent and adult SRS scores demonstrating moderate-to-good temporal reliability [51], the original date of SRS administration was not available in the SPARK data accessible to this study and will be treated as a limitation.

#### PROMIS-PF Short Form

The 8-item PROMIS-PF short form assesses participants’ self-reported ability to perform physical activities. Developed through rigorous psychometric methods and supported by the NIH, PROMIS measures are validated across diverse populations and health conditions, including cognitive impairment and depression [42,43]. PROMIS-PF provides a standardized, patient-centered measure of functional mobility and is available in multiple languages to accommodate participant preferences. PROMIS will provide a unique added value by providing a measure of patients’ perception of their illness and is useful to clinicians and researchers when attempting to determine factors that may influence health at the level of the individual. PROMIS measures are the result of an initiative supported by the US government through the National Institutes of Health (NIH) to provide psychometrically sound tools for clinical and research purposes with the intent of providing a universal, standardized means of collecting patient-reported information. Furthermore, PROMIS has been demonstrated to be valid and reliable for patients across multiple ethnic and age groups [52] and in a variety of health conditions, including depression [53] and mild cognitive impairment [54]. Incorporating these types of outcome measures into autism research provides innovative means of examining socioenvironmental variables that may influence mobility and how these factors are represented in underserved populations.

#### PROMIS-Global Health

The PROMIS Global Health measure includes 10 items that assess key domains of overall health and well-being [55]. These domains include general health, physical function, pain, fatigue, emotional distress, and social satisfaction. The measure yields 2 summary scores: Global Physical Health (GPH) and Global Mental Health (GMH), both standardized as T-scores based on the US general population. This brief, validated tool is well-suited for remote administration and provides a comprehensive snapshot of participants’ self-reported health status.

#### PROMIS-Instrumental Support

PROMIS-Instrumental Support (PROMIS-IS) will be used to assess perceived social support and interpersonal relationships. The PROMIS measures are scored using T-scores for comparison with the large sample mean corresponding to the development of these measures, with higher scores indicating greater satisfaction or fewer limitations [45].

#### Lower Extremity Functional Scale

The Lower Extremity Functional Scale (LEFS) is a 20-item self-report questionnaire designed to evaluate the functional status of individuals with musculoskeletal conditions affecting the lower limbs [40]. Each item assesses the perceived difficulty in performing common physical activities such as walking, climbing stairs, and engaging in recreational tasks. Responses are scored on a 5-point Likert scale ranging from 0 (extreme difficulty or unable to perform activity) to 4 (no difficulty), with total scores ranging from 0 to 80. Higher scores indicate better lower-extremity function. The LEFS has demonstrated strong psychometric properties, including high reliability, validity, and responsiveness, in both clinical and research settings [40]. However, more recent research has raised concerns about the scale’s content validity, particularly its ability to comprehensively capture all relevant aspects of mobility and function across diverse populations [41]. Considering these limitations, an additional functional survey was incorporated into the protocol to further assess the appropriateness and validity of the LEFS for use in adults with ASD, a population with unique motor and sensory profiles that may not be fully represented by traditional musculoskeletal outcome measures.

#### Fall Risk Scale Assessment Scale

Fall risk was assessed using the US Centers for Disease Control and Prevention (CDC) Stopping Elderly Accidents, Deaths, and Injuries (STEADI) initiative screening questionnaire, a validated clinical tool designed to identify individuals at risk for falls [39,56,57]. The STEADI screener considers multiple contributing factors, including mobility and physical function (eg, balance, gait, strength), medical history (eg, chronic conditions, sensory impairments, and medication use), environmental risks (eg, home safety and assistive device use), and cognitive and mental health status (eg, confusion, depression, and anxiety). Consistent with CDC guidance, total scores were used to stratify fall risk. In this study, a cutoff score of ≥4 was applied to classify participants as having a high fall risk. This threshold has been associated with an increased likelihood of future falls and is commonly used to guide clinical decision-making and fall prevention strategies. The STEADI screening approach is widely implemented in both clinical and community settings and supports the early identification of at-risk individuals, enabling targeted interventions to reduce fall-related morbidity [57,58]. Although not specifically validated in adults with ASD, the screener targets core domains (eg, balance, mobility, fall history, and cognitive factors) that are recognized contributors to fall risk across diverse populations, including individuals with neurodevelopmental and cognitive conditions.

#### Comorbidity Index

The comorbidity burden will be assessed using the weighted Functional Comorbidity Index (w-FCI), a validated tool designed to evaluate the impact of chronic health conditions on functional outcomes in older adults [46]. The w-FCI is an enhancement of the original FCI, which includes 18 conditions scored dichotomously (present/absent). To improve sensitivity to functional impairment, the w-FCI incorporates severity weighting for each condition and includes dementia, a key contributor to functional decline that was omitted in the original index. Each condition will be rated based on its current impact on the participants’ functional status, allowing for a more nuanced assessment of comorbidity. The total score will reflect both the number and severity of conditions, providing a more accurate predictor of rehabilitation outcomes, such as mobility and independence in activities of daily living. The index was selected for its demonstrated predictive validity in geriatric populations and its relevance to functional recovery trajectories.

#### GSLT-PAQ

This self-report survey will be used to assess perceived PA participation [47].

Increasing total scores in the GSLT are associated with increased exercise behavior. Participants’ total GSLT scores can determine activity classification as either active or insufficiently active. Corresponding to the American College of Sports Medicine PA guidelines, 24 units or more are classified as active (meeting PA guidelines), 14-23 units as moderately active (not meeting PA guidelines), and 13 units or fewer as insufficiently active [47]. See Figure 2 for the sequence of outcome measures.

### Statistical Data Analysis Plan

All analyses will be conducted using 2-sided tests with a significance level of α=.05. Descriptive statistics will summarize participant characteristics and study variables. Continuous variables will be summarized using means and standard deviations or medians and interquartile ranges, as appropriate, and categorical variables using frequencies and percentages. Prior to inferential analyses, assumptions of normality, linearity, homoscedasticity, and absence of multicollinearity will be evaluated. If the assumptions for parametric analyses are not met, appropriate nonparametric or robust analytic methods will be applied.

#### Aim 1: Construct Validity and Reliability of PROMIS-PF

Construct validity of the PROMIS-PF will be examined using a hypothesis-testing framework with related indicators of health and physical functioning consistent with COSMIN (Consensus-Based Standards for the Selection of Health Measurement Instrument) recommendations [59,60]. Predefined hypotheses were specified regarding both the expected direction and magnitude of associations. PROMIS-PF scores (higher scores indicate better perceived physical function) are expected to demonstrate:

- Moderate-to-strong positive correlations with other self-reported measures of physical function (eg, LEFS and PROMIS Global Health; r≈0.50-0.70)
- Moderate positive correlations with habitual PA (steps/day; r≈0.30-0.50)
- Small-to-moderate negative correlations with comorbidity burden and fall risk indicators (r≈−0.20 to −0.50), where higher scores reflect poorer status.

Associations will be examined using Pearson correlation coefficients when parametric assumptions are met and Spearman rank correlations when assumptions are violated. Correlation coefficients will be reported with 95% CIs, and the interpretation will emphasize agreement with a priori hypotheses [60]. Correlations between PROMIS-PF and other self-report instruments (eg, LEFS and PROMIS Global Health) will be interpreted with caution, as these comparisons are subject to shared method variance, which may inflate associations due to common response tendencies rather than true construct overlap. Accordingly, these analyses will be considered supportive but weaker evidence of convergent validity.

Greater emphasis will be placed on the associations between PROMIS-PF and objective remote performance-based measures, which provide stronger evidence of construct validity due to reduced susceptibility to shared measurement bias. However, these comparisons are limited to a subset of 25 participants and will be interpreted as exploratory. These remote performance-based measures include the 5xSTS, the 30sSTS, and the Standing SLS test under EO and EC conditions. PROMIS-PF scores are expected to correlate negatively with 5xSTS completion time (r ≈ −0.40 to −0.70) and positively with 30sSTS repetitions and SLS duration (r≈0.40-0.70), such that better self-reported function corresponds to better observed functional performance and balance [44,60]. Given the smaller subsample, these analyses will be considered exploratory and interpreted with appropriate caution due to wider CIs. Adjustment for multiple comparisons will be applied within related families of analyses as appropriate.

Reliability will be evaluated for rater-derived scores from recorded remote performance assessments (ie, reliability of scoring rather than repeat participant testing). All sessions will be video-recorded and independently scored by 6 raters to estimate interrater reliability. To estimate intrarater reliability, each rater will re-score the same recordings after a 2-5-day interval. Intraclass correlation coefficients (ICCs) with 95% CIs will be calculated using absolute-agreement models appropriate to the rating design. ICC values will be interpreted using standard thresholds: ≥0.90 (excellent), 0.75-<0.90 (good), 0.50-<0.75 (moderate), and <0.50 (poor) [61-64]. We hypothesize that repeated ratings of recorded remote performance assessments will demonstrate strong interrater and intrarater reliability (ICC ≥0.80).

An a priori power analysis was conducted using G*Power (version 3.1.9.7; Heinrich Heine University Düsseldorf) [63]. Assuming a 2-tailed α=.05 and power=0.80, a total sample size of N=155 provides adequate power to detect small-to-moderate correlations (r ≥ 0.25-0.30), consistent with expected construct validity effect sizes. PROMIS-PF scores are expected to demonstrate moderate positive correlations with habitual PA and performance-based functional measures and moderate-to-strong positive correlations with other self-reported function scales. Negative correlations are anticipated with fall risk indicators and comorbidity burden. In addition to the power-based justification, precision was considered. At N=155, correlation estimates are expected to have reasonably narrow 95% CIs, supporting adequate precision in estimating associations [64].

The sample size for reliability analyses was informed by Borg et al [59]. Assuming 2 repeated measurements per rater, a subsample of n=25 is expected to provide sufficient precision to estimate ICC values ≥0.75, consistent with recommendations for reliability studies.

The full sample (N=155) will be used for primary construct validity analyses, while the nested subset (n=25) will support exploratory performance-based validity analyses and reliability estimation.

#### Aim 2: Racial and Ethnic Disparities in Physical Function, PA, and QoL

To examine racial and ethnic disparities in physical function, PA, personal and environmental factors, and QoL among adults with ASD, analysis of covariance models will be used to compare adjusted mean outcomes between non-Hispanic White participants and participants from racial and ethnic minoritized groups. Covariates such as sex and autism severity will be included as appropriate to reduce confounding. If analyses are expanded to include more than 2 racial and ethnic groups, omnibus models will first be conducted, followed by multiplicity-adjusted pairwise comparisons where the overall test is statistically significant. Effect sizes and 95% CIs will be reported alongside *P* values. The planned sample size of 155 participants is expected to provide adequate power to detect medium-sized effects and to generate preliminary disparity estimates for future studies [63].

#### Aim 3: Associations of Physical Function, PA, Personal and Environmental Factors, and QoL, and Moderation by Race and Ethnicity

To examine the associations among physical function, PA, personal and environmental factors, and QoL, multiple linear regression analyses will be performed with QoL as the dependent variable. The primary independent variables will include PROMIS-PF and habitual PA (steps per day), with additional personal and environmental factors entered as indicated by the conceptual model. Covariates such as sex and autism severity will be included as appropriate. We hypothesize that better physical function and greater PA will be associated with better QoL. To evaluate moderation by race and ethnicity, interaction terms between race/ethnicity and physical function, as well as between race/ethnicity and PA, will be entered into the regression models. Continuous predictors will be centered prior to the creation of interaction terms to improve interpretability and reduce multicollinearity. When significant interactions are identified, simple slopes or stratified analyses will be used to further characterize the nature of the moderation effect. Model results will be reported using unstandardized and standardized regression coefficients, 95% CIs, *P* values, and model fit statistics (eg, adjusted *R*^2^). The same a priori power analysis described above applies to aim 3; although this pilot study may be underpowered to detect small effects, it is expected to provide preliminary effect size estimates to guide future research [63].

#### Missing Data

The extent and pattern of missing data will be examined before analysis, including missingness arising from incomplete wearable monitoring, partial remote assessment completion, technical disruptions during telehealth sessions, and unavailable registry-derived variables. If missing data are minimal, complete-case analyses will be used. If missing data are more substantial and judged to be consistent with a missing-at-random mechanism, multiple imputation may be considered as a sensitivity analysis for key multivariable models. Measure-specific completion rules (eg, Fitbit wear-time thresholds) will be applied before analytic inclusion. Any deviations from the primary analytic plan will be documented and justified.

## Results

### Overview

**Figure 3:**
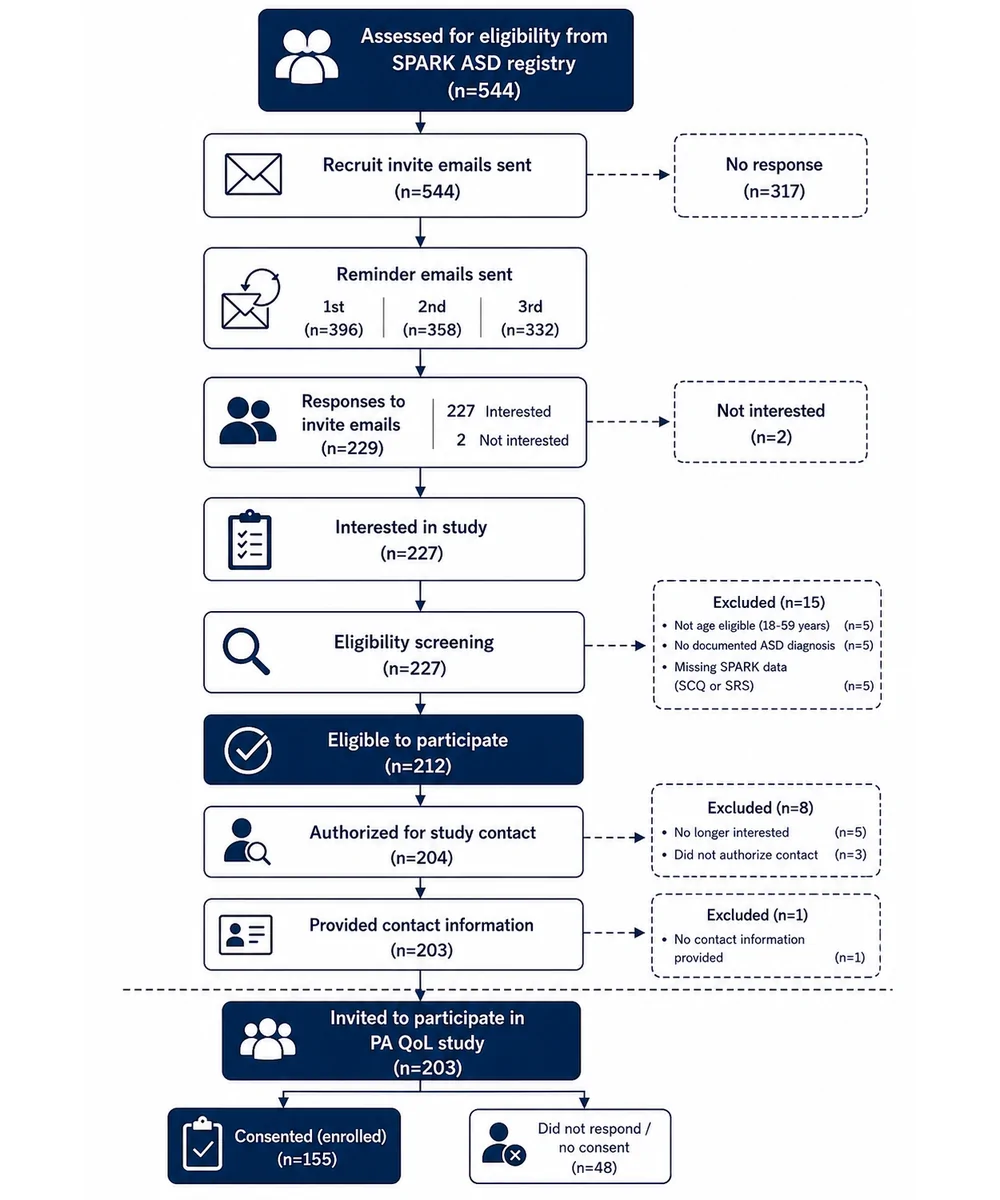
Flow diagram of participant recruitment, eligibility, screening, and enrollment. ASD: autism spectrum disorder; PA: physical activity; QoL: quality of life; SPARK: Simons Powering Autism Research and Knowledge; SRS: Social Responsiveness Scale.

Funding for this study was active from August 2024 through May 2025. Participant recruitment was conducted from December 2024 to March 2025, and final data collection was completed in May 2025. As of May 2025, 155 participants had been recruited into the study. Figure 3 summarizes participant recruitment and enrollment. Data analysis is projected to be completed by July 2026, and the primary study results are anticipated to be submitted for publication in August 2026.

### Recruitment

Of 544 adults with ASD contacted through the SPARK Registry, 229 responded to invitation emails, and 227 expressed interest in participating (see Figure 3). Following the eligibility screening, 15 individuals were excluded, resulting in 212 eligible participants. Of these, 203 were invited to participate, and 155 ultimately provided electronic informed consent and were enrolled in the study. Among enrolled participants, 101 (65.2%) identified as female, 53 (34.2%) identified as male, and 1 (0.6%) selected rather not to say.

The sample was racially and ethnically diverse: 79 (51.0%) participants identified as Black/African American, 40 (25.8%) as Hispanic, 34 (21.9%) as non-Hispanic White, 1 (0.6%) as Asian, and 1 (0.6%) did not report race/ethnicity. Table 2 presents the participant demographic characteristics.

**Table 2:** Sex and race/ethnicity of enrolled participants (N=155).

Sex	Non-Hispanic White, n (%)	Black/African American, n (%)	Hispanic, n (%)	Asian, n (%)	Did not report, n (%)	Total, n (%)
Male	9 (17.0)	19 (35.8)	25 (47.2)	0 (0.0)	0 (0.0)	53 (34.2)
Female	25 (24.8)	60 (59.4)	14 (13.9)	1 (1.0)	1 (1.0)	101 (65.2)
Rather not to say	0 (0.0)	0 (0.0)	1 (100.0)	0 (0.0)	0 (0.0)	1 (0.6)
Total^a^	34 (21.9)	79 (51.0)	40 (25.8)	1 (0.6)	1 (0.6)	155 (100.0)

^a^Values in the total row and column are presented as n (% of the total sample). Percentages may not sum to 100 because of rounding.

### Analysis Status

Data analysis is projected to be completed by July 2026. Submission of the primary results manuscript is anticipated in August 2026.

## Discussion

### Anticipated Principal Findings

This study is expected to generate preliminary evidence regarding the feasibility, validity, and equity relevance of remotely administered physical function and balance assessments in adults with ASD. If the planned hypotheses are supported, the study will demonstrate that patient-reported physical function is associated in the expected direction with related indicators of health and physical functioning, including PA, fall risk, lower-extremity functional outcomes, and comorbidity burden. The study is also expected to provide preliminary evidence regarding the reliability of rater scoring of recorded remote performance tasks and the feasibility of telehealth-delivered physical function assessments in adults with ASD.

### Comparison With Prior Work and Conceptual Rationale

This protocol represents, to our knowledge, the first attempt to systematically evaluate the reliability and validity of remote, performance-based physical function and balance assessments in adults with ASD. Prior work in other populations supports the relevance of combining patient-reported and performance-based measures. More recently, moderate-to-strong associations were demonstrated between PROMIS-PF and common performance-based measures such as the 5xSTS, Time Up and Go, and 10-Meter Walk Test in outpatient neurologic rehabilitation, highlighting the complementary value of these approaches [44]. However, that study was conducted using retrospective, in-clinic data and did not address feasibility, reliability, or validity in remote assessment contexts or in autistic populations [44]. By adapting standardized assessments for telehealth delivery, the present study addresses an important gap in rehabilitation research for neurodivergent populations and applies a prospective remote-assessment approach. Persistent motor impairments, including deficits in coordination, balance, and gait, remain underresearched in adults with ASD despite their relevance to fall risk, reduced PA, and diminished participation [65]. The protocol is grounded in the MHDD framework, which emphasizes the interaction among health conditions, functional status, activity, participation, and contextual factors in shaping QoL [25]. Within this framework, remote physical function assessment is positioned as a practical approach for identifying modifiable factors that may influence participation, PA, and health equity. Findings from this work are expected to help address the need for standardized, accessible, and psychometrically sound functional assessments that remain relevant across adulthood in ASD [66].

### Clinical and Research Implications

If the planned assessments are found to be feasible and psychometrically supported, this protocol has the potential to inform new models of physical therapy assessment for adults with ASD by enabling clinicians to administer standardized functional measures remotely. Such an approach may reduce barriers associated with transportation, sensory sensitivities, and anxiety, all of which can limit access to in-person services for adults with ASD [67,68]. These barriers may also contribute to reduced PA and increased fall risk. Telehealth-based assessment, combined with wearable activity monitoring, may therefore improve participation in both research and clinical care among individuals who might otherwise be underrepresented or unable to access physical therapy services. In addition, examining the relationship between remotely observed performance and patient-reported outcomes may strengthen clinical interpretation and support more individualized care planning.

### Advancing Health Equity

A central contribution of this protocol is its focus on health equity. Telehealth delivery aligns with broader efforts to reduce health care disparities among adults with ASD, particularly among racial and ethnic minority groups who often face additional structural barriers to care [69,70]. By evaluating the reliability and validity of remote physical function assessments, this study may help establish a foundation for more scalable and equitable rehabilitation strategies. Remote assessment may be especially beneficial for adults with ASD who experience anxiety, sensory overresponsiveness, or transportation barriers that make clinic-based participation difficult. Conducting assessments within the home environment may reduce these barriers by allowing participation in a setting that is familiar, controlled, and often more tolerable. If the proposed measures demonstrate acceptable performance, they may support more equitable access to observational physical assessments and provide clinicians with alternatives to relying exclusively on questionnaires. This work may also inform future intervention studies aimed at improving PA, physical functioning, and QoL in diverse adult ASD populations.

### Strengths and Limitations

This protocol has several strengths. First, it integrates patient-reported, behavioral, and remotely observed indicators of physical function into a single telehealth-based study design. Second, it evaluates both construct validity and rating reliability, thereby strengthening the measurement framework for remote assessments in adults with ASD. Third, the protocol explicitly incorporates racial and ethnic disparities in physical function, PA, and QoL, which may inform future equity-focused research and intervention.

Several limitations should also be acknowledged. As a pilot study, the project may be underpowered to detect small effects, particularly in subgroup and moderation analyses. In addition, the sample includes independently living adults with relatively high cognitive abilities, which may limit generalizability to individuals with greater support needs. A further limitation is the overrepresentation of females in the SPARK registry relative to historical ASD diagnosis ratios, although more recent literature suggests that previous estimates of the male-to-female ratio may partly reflect underdiagnosis or delayed diagnosis in females [71-73]. Future studies should prioritize broader representation across sex, racial and ethnic background, cognitive profiles, and levels of required support. Finally, remote performance-based assessments may be influenced by the home environment, camera positioning, technology access, and participant familiarity with telehealth procedures. Accordingly, the primary contribution of this study is expected to be methodological and hypothesis-generating rather than definitive.

### Future Directions

This protocol may provide a foundation for integrating telehealth into neurodiverse rehabilitation research and practices. If the planned psychometric and feasibility findings are supported, future studies should replicate these methods in larger and more heterogeneous cohorts, including a greater representation of males, individuals with varying cognitive abilities, and adults with greater support needs. Future studies should also examine the longitudinal relationships between anxiety, perceived fall risk, physical performance, and PA in adults with ASD. In addition, evaluating whether race and ethnicity moderate the relationships among physical function, PA, and QoL may yield important insights for developing more tailored and equity-oriented interventions in the future.

### Dissemination Plan

Study findings will be disseminated through peer-reviewed journal publication, conference presentations, and communication with relevant clinical, research, and community stakeholders. The findings from this pilot protocol are also expected to inform future grant applications and the design of larger studies focused on remote physical function assessment, PA, and health equity in adults with ASD.

### Conclusions

This protocol describes a pilot study designed to evaluate the feasibility, validity, and equity of remotely administered physical function and balance assessments in adults with ASD. If the planned hypotheses are supported, this study will provide preliminary evidence to guide the use of telehealth-based physical function measures in future research and may inform more accessible approaches to physical therapy assessment in this population. The anticipated contribution of this work is primarily methodological and foundational, with the goal of supporting larger, fully powered studies that can more definitively examine functional health, participation, and QoL in adults with ASD.

## Data Availability

The data that support the findings of this study are not openly available due to sensitivity reasons and are available from the corresponding author upon reasonable request.

## Abbreviations

30sSTS: 30-Second Sit-to-Stand
5xSTS: Five Times Sit-to-Stand
ASD: autism spectrum disorder
BBS: Berg Balance Scale
CDC: Centers for Disease Control and Prevention
COSMIN: Consensus-Based Standards for the Selection of Health Measurement Instrument
DPT: Doctor of Physical Therapy
EC: eyes close
EO: eyes open
FCI: Functional Comorbidity Index
GMH: Global Mental Health
GPH: Global Physical Health
GSLT-PAQ: Godin-Shephard Leisure-Time Physical Activity Questionnaire
ICC: intraclass correlation coefficient
ICF: International Classification of Functioning, Disability, and Health
LEFS: Lower Extremity Functional Scale
MHDD: Model of Healthcare Disparities and Disability
PA: physical activity
PROMIS: Patient-Reported Outcomes Measurement Information System
PROMIS-PF: Patient-Reported Outcomes Measurement Information System–Physical Function
PT: physical therapist
QoL: quality of life
SFARI: Foundation Autism Research Initiative
SLS-R/L: right/left single-leg stance
SPARK: Simons Powering Autism Research and Knowledge
SRS: Social Responsiveness Scale
STEADI: Stopping Elderly Accidents, Deaths, and Injuries
w-FCI: weighted Functional Comorbidity Index
